# Enhanced Heuristic Drift Elimination with Adaptive Zero-Velocity Detection and Heading Correction Algorithms for Pedestrian Navigation

**DOI:** 10.3390/s20040951

**Published:** 2020-02-11

**Authors:** Ruihui Zhu, Yunjia Wang, Baoguo Yu, Xingli Gan, Haonan Jia, Boyuan Wang

**Affiliations:** 1 Key Laboratory of Land Environment and Disaster Monitoring, MNR, China University of Mining and Technology, Xuzhou 221116, China; zhulucking@163.com; 2 State Key Laboratory of Satellite Navigation System and Equipment Technology, 589 ZhongShan Street, Qiaoxi District, Shijiazhuang 050081, China; zhu_lucking@163.com (B.Y.); ganxingli@163.com (X.G.); jiahaonan1022@163.com (H.J.); boyuan@hrbeu.edu.cn (B.W.)

**Keywords:** pedestrian navigation, adaptive ZUPT, heuristic, complex path, heading correction

## Abstract

As pedestrian dead-reckoning (PDR), based on foot-mounted inertial sensors, suffers from accumulated error in velocity and heading, an improved heuristic drift elimination (iHDE) with a zero-velocity update (ZUPT) algorithm was proposed for simultaneously reducing the error in heading and velocity in complex paths, i.e., with pathways oriented at 45°, curved corridors, and wide areas. However, the iHDE algorithm does not consider the changes in pedestrian movement modes, and it can deteriorate when a pedestrian walks along a straight path without a pre-defined dominant direction. To solve these two problems, we propose enhanced heuristic drift elimination (eHDE) with an adaptive zero-velocity update (AZUPT) algorithm and novel heading correction algorithm. The relationships between the magnitude peaks of the *y*-axis angular rate and the detection thresholds were established only using the readings of the three-axis accelerometer and the three-axis gyroscopic, and a mechanism for constructing temporary dominant directions in real time was introduced. Real experiments were performed and the results showed that the proposed algorithm can improve the still-phase detection accuracy of a pedestrian at different movement motions and outperforms the iHDE algorithm in complex paths with many straight features.

## 1. Introduction

With the development of microelectronics technology, the volume of micro-electro-mechanical systems’ (MEMS) inertial measurement units (IMU) is getting smaller, and the price is getting lower which make them popular for pedestrian dead reckoning (PDR). However, the drift error inherent in the gyroscope and the accumulated error of the accelerometer result in position error accumulation with the running time [1].

Pedestrian navigation trajectories will deviate from the real walking routes with the accumulated errors in velocity and heading. Reducing the errors to a reasonable range is a major challenge for research in pedestrian navigation. To reduce the accumulated error of the accelerometer, the zero velocity update (ZUPT) was used to aid the foot-mounted inertial navigation system (INS) [2,3,4] in the extended Kalman filter (EKF) framework, called the INS-EKF-ZUPT (IEZ), which is effective for suppressing the accumulated error in velocity. When a person walks, the feet are periodically separated (swing-phase) and contacted (still-phase) from the ground [5]. The basic idea of ZUPT is to reset the velocity when the feet are detected as being relatively stationary with the ground. Therefore, the performance of ZUPT highly relies on the still-phase detection accuracy. There are various methods for gait detection based on the outputs of the accelerometer and the gyroscope. According to the different sources of data, the methods for still-phase detection can be divided into three categories: acceleration-based, angular rate-based, and both acceleration and angular rate-based. The gait detection methods using acceleration include amplitude detection [6], moving mean detection [7], and moving variance detection [8], etc. The angular rate is used in the form of amplitude [9], moving mean [10], and root mean square [11]. The methods based on both acceleration and angular rate usually select one or more methods from the above single source methods for logical AND operation [12,13]. The methods mentioned above are all pre-defined threshold-based, and the detectors provide good performance at slow speeds and normal speeds. However, when a person is walking and running, the optimal threshold values could differ significantly. The readings of the inertial measurement unit tend to become larger and the zero velocity intervals tend to become shorter when a person is running. If the pre-defined threshold values are too small, the zero velocity intervals cannot be detected. To solve this problem, extensive research has been performed. Ren et al. [14] proposed a novel pedestrian navigation algorithm for a foot-mounted inertial sensor-based system which adjusts the threshold adaptively using the hidden Markov model (HMM) according to the pedestrian’s motion modes. Zhang et al. [15] proposed an adaptive zero velocity update method based on velocity classification for pedestrian tracking which establishes the mapping between the acceleration of two accelerometers and the detection threshold with one accelerometer mounted on the foot and the other attached to the chest, and the information extracted from the chest acceleration is used to update the corresponding threshold for the still-phase detection. Tian et al. [16] proposed a novel velocity interval detection algorithm based on a smoothed pseudo Wigner–Ville distribution to remove multiple frequencies intelligently (SPWVD-RMFI) which adopts the SPWVD-RMFI method to extract the pedestrian gait frequency and to calculate the optimal zero velocity interval detection threshold in real time by establishing the function relationships between the thresholds and the gait frequency. These adaptive methods mentioned above are all not only effective but also relatively easy for us to implement. Extra non-inertial sensors have been used to aid zero-velocity detection. Ming et al. [17] proposed an adaptive zero-velocity detection algorithm based on multi-sensor fusion which employs the measurements of an accelerometer, gyroscope, and pressure sensor to construct a zero-velocity detector. Zhou et al. [18] used a shoe-embedded RF sensor for motion detection. Wang et al. [19] applied wearable EMG sensors to measure walking strides. But these methods with the assistance of extra non-inertial sensors all require expensive and specialized equipment. The methods of applying wavelet transform to the acceleration are studied by some scholars [20,21,22], but these methods require lots of computational-power and cannot be handled easily by normal Advanced RISC Machines (ARM) processor [15].

The drift error inherent in the gyroscope contains a slowly changing near-DC component which results in accumulated error in attitude, and the error increases continuously and without bound with run time, called “drift” [23]. The IEZ algorithm is unable to estimate the error in heading because it does not get the information of yaw and angular rate on the z-axis. In order to reduce the accumulated error in heading caused by the near-DC component of drift, some heading correction methods were proposed. The method combining particle filter (PF) with post-map matching was used to obtain optimal navigation results [24]. It uses offline map matching which cannot meet the needs of real-time positioning. Zero angular rate update (ZARU) was proposed by Rajagopal [25]. It feeds into EKF with the measured error in the angular rate when the foot is still and has limited ability to reduce error in heading. The heuristic drift elimination (HDE) algorithm, which is a gyro-based heading estimation, was proposed by Borestein and Ojeda [26]. It makes use of the fact that most indoor buildings are rectangular or square and many corridors are straight; four dominant directions are pre-defined according to the orientation of the corridors. When a pedestrian is detected walking straight along one of the four pre-defined dominant directions, the gyro biases will be corrected by HDE algorithm. Considering that the original HDE algorithm can even damage the navigation solution when used in complex buildings, i.e., with curved corridors, pathways oriented other than 90°, or wide areas for non-oriented motion, an improved heuristic drift elimination (iHDE) was proposed which studied the performance of HDE-based methods in complex buildings, i.e., with pathways oriented at 45°, curved corridors, and wide areas where non-oriented motion is possible. Compared with the original HDE implementation, the iHDE algorithm performs very well in ideal orthogonal narrow-corridor buildings and outperforms HDE for non-ideal trajectories [27]. However, the iHDE algorithm can only work in one of eight pre-defined dominant directions, if a pedestrian is walking straight along a straight path without pre-defined dominant direction, it does not work. Indoor pedestrian navigation using an INS/EKF framework for yaw drift reduction was proposed [13] which uses the difference between the stride directions and the pre-defined dominant directions as the measured error in a Kalman Filter framework and combines HDE with IEZ for simultaneously reducing the errors in velocity and heading. Castro-Toscano et al. [28] described a method for tracking the position of a moving object using an inertial navigation system with a Kalman filter (INS/KF) and an implementation of the zero-velocity update and zero angular rate update (ZUPT/ZARUT) algorithms. Its main contribution is the methodological recommendations for integrating INS-KF-ZUPT/ZARUT or IKZ into the re-feed INS strapdown system. A study of mathematical descriptions for inertial navigation systems and integration of virtual sensors implementation is presented in Reference [29] which aims to calculate variables such velocity, position, and attitude on rigid or mobile bodies of navigation systems. Abdulrahim et al. [7] proposed an aiding MEMS IMU with a building heading method for indoor pedestrian navigation which uses imagery-derived building heading to reduce heading drift error. However, the original HDE algorithm and iHDE algorithm mentioned above require four or eight pre-defined dominant directions which is not suitable for all straight path segments in complex trajectory. Besides, the iHDE algorithm, although integrated with ZUPT, cannot adjust the detection threshold adaptively.

Different from the existing works, an enhanced heuristic drift elimination algorithm is proposed in this paper, and the main contributions are summarized as below:

- An adaptive zero velocity update algorithm (AZUPT) is introduced in this paper to improve the still-phase detection accuracy of a pedestrian with different movement motions (walking at normal speed/running slowly), the relationship between magnitude peak of *y*-axis gyroscope and detection threshold is constructed only using the readings of one MEMS-IMU, and the AZUPT algorithm is combined with INS in EKF framework to estimate the error in velocity.
- A novel heading correction algorithm is proposed to make up for the shortcomings of the iHDE algorithm that cannot work in the straight path without dominant direction, and a strict straight walking detection mechanism is introduced to determine whether the pedestrian is walking straight or not.
- An enhanced heuristic drift elimination with adaptive zero-velocity detection algorithm and novel heading correction algorithm is proposed for pedestrian navigation with different movement motions in complex paths. Based on the proposed algorithm, real experiments were carried out to evaluate the performance of the adaptive zero detection algorithm with different people, the performance of the proposed heading correction method, and the performance of eHDE algorithm in complex paths with many straight features.

The remainder of this paper is organized as follows: the materials and methods are described in Section 2; the problems are analyzed in Section 3; Section 4 describes the proposed algorithm; the real experimental research and analysis based on our proposed algorithm are presented in Section 5; Section 6 is the conclusion; and Abbreviations is a table of the abbreviations and initials.

## 2. Materials and Methods

### 2.1. Introduction of the INS-EKF-ZUPT Algorithm

#### 2.1.1. Reference Frames

**Figure 1:**
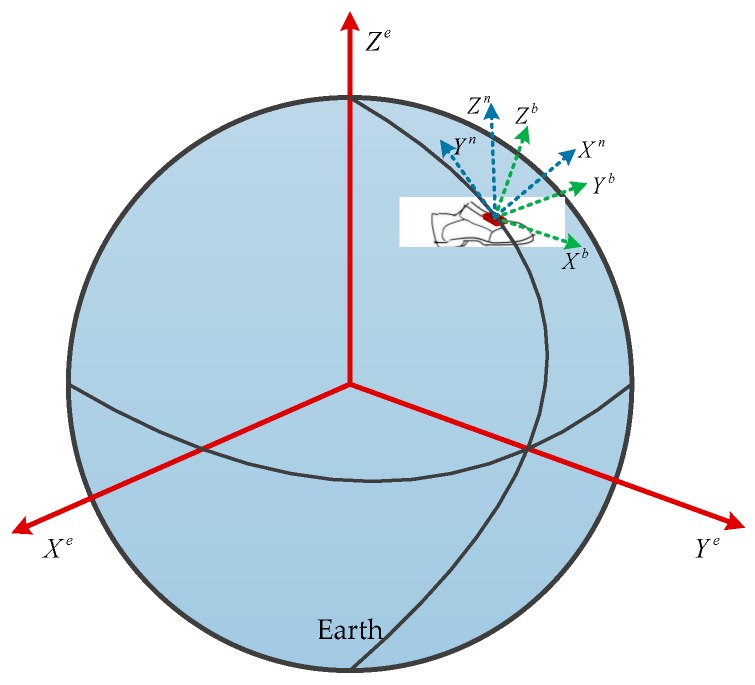
The reference frames.

It is important to define the body frame (*b*) and navigation frame (*n*) in IMU-based pedestrian navigation. Figure 1 shows the definition of the two frames. The origin of the two frames are the MEMS-IMU center of mass. The body frame is defined as the *x*-axis pointing in the forward direction, the *z*-axis pointing up in the vertical direction, and the *y*-axis following the right-handed rule. The navigation frame is defined aligning to local north, east, and up direction.

#### 2.1.2. INS for Position and Attitude Estimation

**Figure 2:**
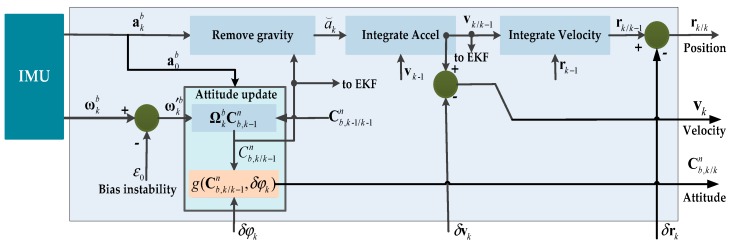
The inertial navigation system (INS) mechanization using the extended Kalman Filter (EKF) framework.

An inertial navigation system is an autonomous navigation system that does not depend on any external information and does not radiate energy to the outside. It has the characteristic of good concealment and can work in various complex environments which make it popular for PDR. The inertial measurement unit used for INS generally includes a three-axis accelerometer and a three-axis gyroscope; it is usually mounted on the body directly. The original readings of the IMU, acceleration $a_{k}^{b}$ and angular rate $\omega_{k}^{b}$, respectively, on the body (*b*) frame, were taken at discrete sampling time *k*. The INS mechanization used in the EKF framework is shown in Figure 2. It was implemented with some modifications to cope with the estimation error state vector: $\delta x_{k}\;=\;\left[\delta \varphi_{k},\delta \omega_{k},\delta r_{k},\delta v_{k},\delta a_{k}^{b}\right]$ in angular, velocity, and position provided by EKF. Every component in $\delta x_{k}$ had three elements, corresponding to the *x*-dimensional, *y*-dimensional, and *z*-dimensional.

A detailed description of the INS mechanization is as follows:

(1) The initial deviation in the angular rate $\varepsilon_{0}$ was removed from angular rate $\omega_{k}^{b}$.

(1) $$\omega_{k}^{\prime b}\;=\;\omega_{k}^{b}-\varepsilon_{0}$$

where $\varepsilon_{0}$ is the average of the angular rate over a period of time (20 s in this paper) since the gyroscope was powered on.

(2) Attitude update.

The initial attitude was calculated using the readings of the three-axis accelerometer on the body frame.

(2) $$\begin{array}{c} Roll_{0}\;=\;\mathrm{atan}\left(\frac{a_{0,z}^{b}}{a_{0,y}^{b}}\right)\\ Pitch_{0}\;=\;\mathrm{atan}\left(\frac{\sqrt{{\left(a_{0,y}^{b}\right)}^{2}+{\left(a_{0,z}^{b}\right)}^{2}}}{a_{0,x}^{b}}\right)\\ Yaw_{0}\;=\;0 \end{array}$$

where $a_{0}^{b}\;=\;(a_{0,x}^{b},a_{0,y}^{b},a_{0,z}^{b})$ is the acceleration for calculating the initial attitude.

The initial rotation matrix $C_{b,0}^{n}$ which transforms from the body (*b*) frame to the navigation (*n*) frame is given as:

(3) $$C_{b,0}^{n}\;=\;C_{2,0}^{n}C_{1,0}^{2}C_{b,0}^{1}$$

where

(4) $$C_{b,0}^{1}\;=\;\left[\begin{array}{ccc} 1 & 0 & 0\\ 0 & \cos\left(Roll_{0}\right) & -\sin\left(Roll_{0}\right)\\ 0 & \sin\left(Roll_{0}\right) & \cos\left(Roll_{0}\right) \end{array}\right]$$

(5) $$C_{1,0}^{2}\;=\;\left[\begin{array}{ccc} \cos\left(Pitch_{0}\right) & 0 & \sin\left(Pitch_{0}\right)\\ 0 & 1 & 0\\ -\sin\left(Pitch_{0}\right) & 0 & \cos\left(Pitch_{0}\right) \end{array}\right]$$

(6) $$C_{2,0}^{n}\;=\;\left[\begin{array}{ccc} \cos\left(Yaw_{0}\right) & -\sin\left(Yaw_{0}\right) & 0\\ \sin\left(Yaw_{0}\right) & \cos\left(Yaw_{0}\right) & 0\\ 0 & 0 & 1 \end{array}\right]$$

The initial rotation matrix was calculated using the acceleration, and the rotation matrix $C_{b,k}^{n}$ was updated with gyroscopic information at epoch *k*:

(7) $$C_{b,k/k-1}^{n}\;=\;\Omega_{k}^{b}C_{b,k-1/k-1}^{n}$$

where $C_{b,k-1/k-1}^{n}$ is the rotation matrix corrected by EKF at epoch *k* − 1. The $\Omega_{k}^{b}$ is the skew symmetric matrix for angular rates at epoch *k*:

(8) $$\Omega_{k}^{b}\;=\;\left[\begin{array}{ccc} 0 & -\omega_{k,z}^{\prime b} & \omega_{k,y}^{\prime b}\\ \omega_{k,z}^{\prime b} & 0 & -\omega_{k,x}^{\prime b}\\ -\omega_{k,y}^{\prime b} & \omega_{k,x}^{\prime b} & 0 \end{array}\right]$$

The rotation matrix $C_{b,k/k-1}^{n}$ calculated previously was refined with the three-axis angle errors estimated by EKF. Assuming that the three-axis angle errors are small, the corrected rotation matrix $C_{b,k}^{n}$ can be computed using another Pade’s approximation, as [13]:

(9) $$C_{b,k}^{n}\;=\;g\left(C_{b,k/k-1}^{n},\delta \varphi_{k}\right)\;=\;\frac{2I_{3\times 3}+\delta \Theta_{k}}{2I_{3\times 3}-\delta \Theta_{k}}\cdot C_{b,k/k-1}^{n}$$

where $\delta \Theta_{k}$ is the skew symmetric matrix for small angles.

(10) $$\delta \Theta_{k}\;=\;-\left[\begin{array}{ccc} 0 & -\delta \varphi_{k}\left(3\right) & \delta \varphi_{k}\left(2\right)\\ \delta \varphi_{k}\left(3\right) & 0 & -\delta \varphi_{k}\left(1\right)\\ -\delta \varphi_{k}\left(2\right) & \delta \varphi_{k}\left(1\right) & 0 \end{array}\right]$$

(3) Firstly, the raw acceleration was transformed from the body frame (*b*) to the navigation frame (*b*), and then the gravity, g, was subtracted from the vertical component of the acceleration.

(11) $${\overset{ˇ}{a}}_{k}\;=\;C_{b,k/k}^{n}a_{k}^{b}-\left[\begin{array}{ccc} 0 & 0 & g \end{array}\right]$$

where ${\overset{ˇ}{a}}_{k}$ is the acceleration under the navigation frame (*n*) without the gravity component.

(4) The acceleration ${\overset{ˇ}{a}}_{k}$ was integrated to get the velocity $v_{k/k-1}$ in the navigation frame n, and in a second integration, the position $r_{k/k-1}$.

(12) $$\{\begin{array}{l} v_{k/k-1}\;=\;v_{k-1}+{\overset{ˇ}{a}}_{k}\cdot \Delta t\\ r_{k/k-1}\;=\;r_{k-1}+v_{k/k-1}\cdot \Delta t \end{array}$$

(5) Position and velocity computed previously were updated once the measured error was estimated by EKF at epoch *k*:

(13) $$\{\begin{array}{c} v_{k}\;=\;v_{k-1/k}-\delta v_{k}\\ r_{k}\;=\;r_{k-1/k}-\delta r_{k} \end{array}$$

#### 2.1.3. Still Phase Detection

The performance of the zero-velocity update algorithm highly relies on still-phase detection accuracy. Most methods for still-phase detection use the signal processing technique with the readings of accelerometers or gyroscopes [15,17,30]. The local acceleration standard deviation-based methods are commonly used for still-phase detection; if the local acceleration standard deviation is below the given threshold, it is determined to be still-phase or it is swing phase.

The conditions to declare a foot in still-phase are derived as below based on the readings of accelerometers.

(14) $$\{\begin{array}{ll} T_{k}\;=\;\sqrt{\frac{1}{W}\sum_{k\;=\;i-w}^{k\;=\;i+w}\left(a_{k}-{\bar{a}}_{i}\right)}<T_{th}, & still-phase\\ others, & swing-phase \end{array}$$

where

(15) $$a_{k}\;=\;\sqrt{a_{k,x}^{2}+a_{k,y}^{2}+a_{k,z}^{2}}$$

(16) $${\bar{a}}_{i}\;=\;\frac{1}{W}\overset{k\;=\;i+w}{\underset{k\;=\;i-w}{\sum }}a_{k}$$

(17) $$W\;=\;\frac{1}{2w+1}$$

where *W* denotes the size of the window; $T_{k}$ denotes the test statistic at epoch *k*; $a_{k}\;=\;\left(a_{k,x},a_{k,y},a_{k,z}\right)$ is acceleration at epoch *k*; $T_{th}$ is the pre-defined fixed threshold.

#### 2.1.4. The Measured Error in Velocity

When the foot is detected relatively stationary with the ground, the velocity derived from INS without correction by EKF is used as the measurements of the measured error in the velocity.

(18) $$\Delta v_{k}\;=\;v_{k}-\left[0,0,0\right]$$

### 2.2. Introduction of the Improved Heuristic Drift Elimination Algorithm

The iHDE algorithm studies the performance of HDE-based methods in complex buildings, i.e., with pathways also oriented at 45°, curved corridors, and wide areas where non-oriented motion is possible and eight dominant directions are pre-defined to constrain pedestrian heading. Comparing with the original HDE implementation, the iHDE performs very well in ideal orthogonal narrow-corridor buildings, and iHDE outperforms HDE for non-ideal trajectories. The iHDE algorithm for calculating the error in heading could be summarized in three steps [27]:

Step1: Stride direction: The stride direction of a pedestrian is calculated as:

(19) $$\theta_{s}\left(k\right)\;=\;\arctan\left(\frac{P_{y}^{k}-P_{y}^{k-1}}{P_{x}^{k}-P_{x}^{k-1}}\right)$$

where ($P_{x}^{k},P_{y}^{k}$) is the position calculated by IEZ at epoch *k*.

Step 2: Straight-line path detection (SLPD), more than two user strides are used to judge whether the pedestrian walks straight or not. There is a binary straight-line judgment parameter:

(20) $$\mathrm{SLPD}\left(k\right)\;=\;\{\begin{array}{cc} 1 & \max\left(\left|\theta_{s}\left(j\right)-mean\left(\theta_{s}\left(j\right)\right)\right|\right)<Th_{\theta }\\ & for\;j\;=\;k:k-n\;(n>2)\\ 0 & otherwise \end{array}$$

where $Th_{\theta }$ is an angular threshold and the iHDE works when SLPD is true.

Step 3: The error in heading

The difference between the stride direction and the closest dominant direction, the error in heading, is computed as:

(21) $$\delta \theta_{k}\;=\;\theta_{s,k}-\theta_{b,k}$$

Then, the $\delta \theta_{k}$ is feed into EKF to estimate the error in heading.

### 2.3. Extended Kalman Filter

The error state vector at epoch *k* is:

(22) $$\delta x_{k}\;=\;\left[\delta \varphi_{k},\delta \omega_{k},\delta r_{k},\delta v_{k},\delta a_{k}^{b}\right]$$

The state transition matrix that is a non-linear function in PDR navigation is linearized as:

(23) $$\Phi_{k}\;=\;\left[\begin{array}{ccccc} I & \Delta t\cdot C_{b,k/k}^{n} & 0 & 0 & 0\\ 0 & I & 0 & 0 & 0\\ 0 & 0 & I & \Delta t\cdot I & 0\\ 0 & 0 & 0 & I & \Delta t\cdot C_{b,k/k}^{n}\\ 0 & 0 & 0 & 0 & I \end{array}\right]$$

The state transition model function is:

(24) $$\delta x_{k+1/k}\;=\;\Phi_{k}\delta x_{k/k}+w_{k}$$

where the $\Phi_{k}$ is the state transition matrix; $w_{k}$ is the process noise, its covariance matrix is $Q_{k\;}\;=\;E(w_{k}w_{k}^{T})$ initialized as a diagonal 15 × 15 matrix with these in-diagonal elements: $\left[\begin{array}{cc} \begin{array}{cc} 1\cdot 10_{1\times 3}^{-4} & 0_{1\times 3} \end{array} & \begin{array}{cc} 0_{1\times 3} & \begin{array}{cc} 1\cdot 10_{1\times 3}^{-4} & 0_{1\times 3} \end{array} \end{array} \end{array}\right]$.

The measurement model function is:

(25) $$z_{k+1}\;=\;H\delta x_{k+1/k}+n_{k+1}$$

(26) $$H\;=\;\left[\begin{array}{ccccc} I & 0 & 0 & 0 & 0\\ 0 & 0 & 0 & I & 0 \end{array}\right]$$

where $z_{k+1}$ is the measurement; **H** is the measurement matrix; $n_{k+1}$ is the measurement noise, its covariance matrix is $R_{k+1}\;=\;E\left(n_{k+1}n_{k+1}^{T}\right)$. **R** is a square matrix and we set this matrix with in-diagonal elements with values of: 0.01 for ZUPT and 0.01 for HDR.

The error state vector is updated as:

(27) $$\delta x_{k+1}\;=\;\delta x_{k+1/k}+K_{k+1}\cdot \left[m_{k+1}-H\delta x_{k+1/k}\right]$$

where $m_{k}\;=\;\left[\Delta \varphi_{k},\Delta v_{k+1}\right]$ is the actual error measurement, $K_{k+1}$ is Kalman filter gain that is given as:

(28) $$K_{k+1}\;=\;P_{k+1/k}H^{T}{\left(HP_{k+1/k}H^{T}+R_{k+1}\right)}^{-1}$$

where $P_{k+1/k}$ is the prediction error covariance matrix calculated as below at epoch *k*+1.

(29) $$P_{k+1/k}\;=\;\Phi_{k}P_{k/k}\Phi_{k}^{T}+Q_{k}$$

The error covariance matrix $P_{k+1}$ is computed as:

(30) $$P_{k+1}\;=\;\left(I-K_{k+1}H\right)P_{k+1/k}{\left(I-K_{k+1}H\right)}^{T}+K_{k+1}R_{k+1}K_{k+1}^{T})$$

## 3. The Problems Description

The iHDE algorithm could reduce the cumulated error in velocity and heading. However, on the one hand, the iHDE algorithm does not consider the changes of a pedestrian’s movement modes, and it can be deteriorated when a pedestrian walks with different movement motions. On the other hand, the heading correction part of the iHDE algorithm can only works when a pedestrian is walking along one of the four or eight pre-defined dominant directions. Even if the pedestrian walks straightly along a straight path without a pre-defined dominant direction, it does not work.

### 3.1. The Still-Phase Detection

**Figure 3:**
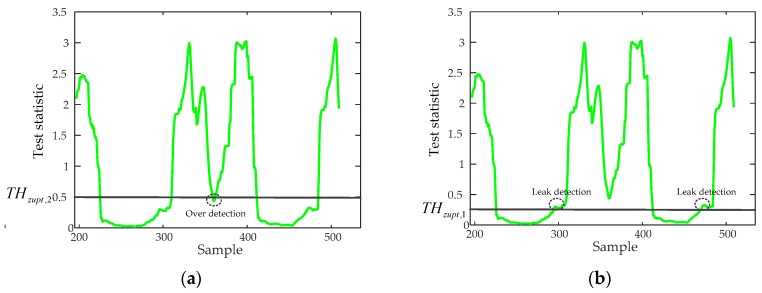
The detection results of still-phase based on the pre-defined threshold method. (**a**) The detection result using a larger threshold; (**b**) the detection result using a smaller threshold.

The performance of the ZUPT algorithm highly relies on the zero-velocity interval’s detection accuracy. A fixed threshold-based still-phase detection algorithm is commonly used to do this work as shown in Equations (14)–(17). The pre-defined threshold $T_{th}$ is an important factor affecting the zero-velocity interval’s detection accuracy. If we use a small threshold $TH_{zupt,1}$, it will lead to the still-phase leakage detection, but if we use a large threshold $TH_{zupt,2}$, it will lead to the still-phase over-detection as shown in Figure 3.

**Figure 4:**
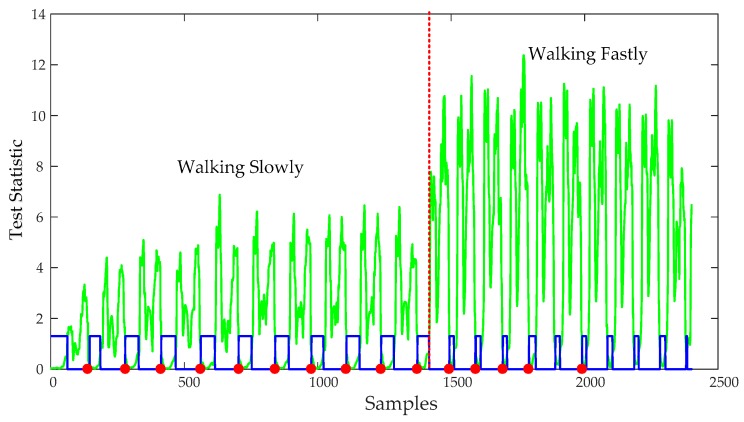
The zero-velocity interval’s detection results for the fixed threshold-based detection algorithm when a pedestrian walks at different speeds. The blue line denotes the gait, the large values indicate the still-phase, while the small values indicate the swing-phase; the red dots denote the start point of the still-phase; and the green line denotes the standard deviation of acceleration.

A reasonable pre-defined threshold can improve the still-phase detection accuracy. However, the movement modes of a pedestrian are diverse, if a person walks slowly, the zero velocity interval lasts a longer time and the still-phase can be detected using a smaller threshold $TH_{zupt,1}$, but if the walking speed of the pedestrian increases, the zero velocity interval becomes smaller and the test statistics becomes larger. If the $TH_{zupt,1}$ is still used to detect the still-phase, it will lead to the still-phase leakage detection as shown in Figure 4. But if we use a larger pre-defined threshold, it may lead to over-detection of still-phase as shown in Figure 3a. Therefore, when a pedestrian walks in different speed, the fixed threshold-based detection algorithms result in many feet steps and still-phase leakage detection. ZUPT algorithm cannot work during the missing detected still-phase, which results in a large position error. Although it is possible to increase the detection accuracy of the still-phase by increasing the pre-defined threshold, it will lead to still-phase over-detection. Therefore, it is necessary for us to introduce an adaptive ZUPT algorithm that can adjust the threshold according to the walking speed for improving the accuracy of still-phase detection.

### 3.2. The Heuristic Drift Elimination and its Improved Algorithm

**Figure 5:**
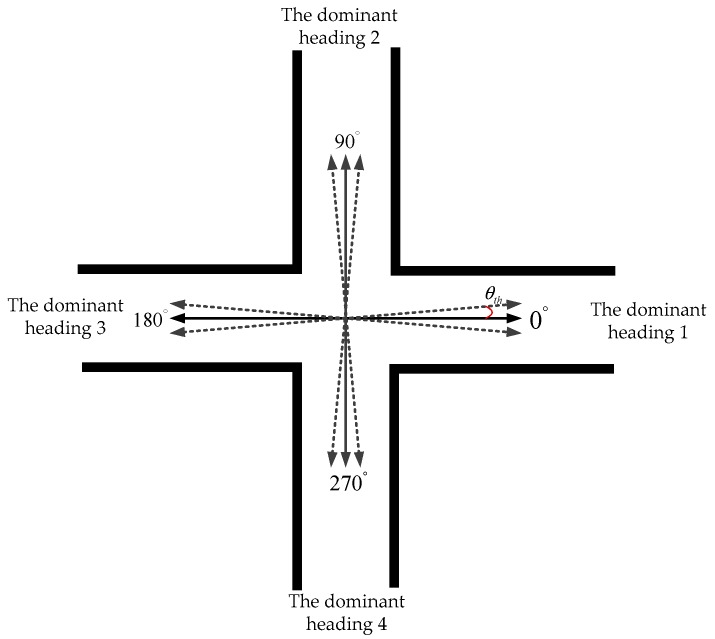
Four dominant directions are pre-defined with the fact that most corridors in buildings are straight and so are most walls and sidewalks alongside which a person might walk.

The original HDE algorithm aims to reduce the accumulated error in heading using only a body-attached IMU; it makes use of the fact that most corridors in buildings are straight and so are most walls and sidewalks alongside which a person might walk, and an I-control is used for correcting the gyro signals when the algorithm assesses that the user is walking along a straight line [23]. Instead of filtering the gyro signals with a binary I-controller, Jiménez et al. [13,29] worked in the yaw space. The original HDE algorithm pre-defined four dominant directions before implementation as shown in Figure 5. When the difference between a pedestrian’s walking heading and the dominant direction *n* is less than the pre-defined threshold $\theta_{th}$, it can be determined that the pedestrian is walking along the dominant direction *n*, and the difference is used as the measured error to update the EKF.

Jimenez et al. [29] pointed out that if a pedestrian walks along the non-dominant directions, HDE algorithm will fail. Although iHDE performs very well in ideal orthogonal narrow-corridor buildings, and outperforms HDE for non-ideal trajectories, iHDE can only work at one of eight pre-defined dominant directions. Even if a pedestrian is walking straight along a straight path with non-dominant direction, it does not work.

## 4. The Proposed Algorithm

**Figure 6:**
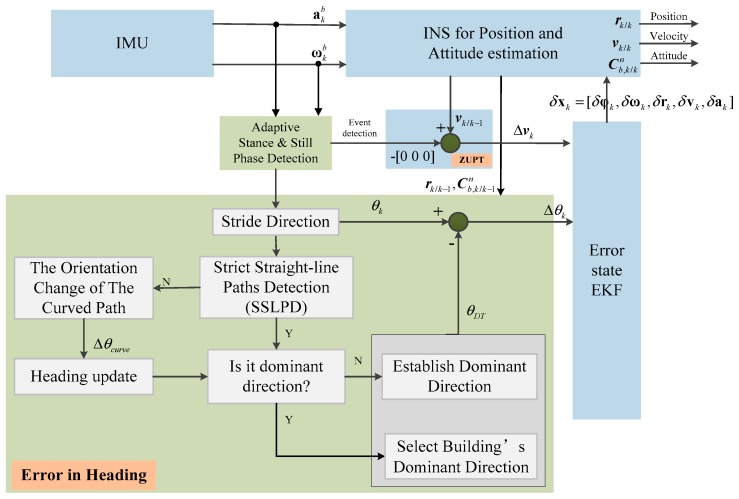
The enhanced Heuristic Drift Elimination (eHDE) algorithm mechanism.

An enhanced Heuristic Drift Elimination algorithm is proposed in this paper, which includes two key technologies compared to iHDE algorithm. One is an adaptive still-phase detection technology. The other is a novel heading correction algorithm which can work in non-dominant direction. Figure 6 shows the mechanism of eHDE algorithm.

### 4.1. Adaptive Still-phase Detection

An adaptive still-phase detection algorithm is introduced in this paper, the relationship between the magnitude peaks of *y*-axis angular rate and the detection threshold is established, although it is similar to [17] implementation only using MEMS IMU. In order to establish the relationship, we conducted six sub-experiments with six different motion modes using a treadmill and the average magnitude peaks of the *y*-axis angular rate were approximately 1.96, 2.82, 3.68, 4.50, 5.0 and 5.5 rad/s respectively. The MEMS-IMU model selected in this paper is XSENS MTI-10-2A5G4-DK (Holland) [31] including three-axis accelerometers and three-axis angular rate meters, the sampling frequency is 100 Hz.

For different walking speeds, the still-phases can be detected using the thresholds as given in Table 1.

According to the results in Table 1, we determined the threshold function using second-order polynomial fitting as follow:

(31) $$f\left(\omega \right)\;=\;0.1178\omega^{2}-0.54\omega +0.762$$

where f(ω) represents the threshold function and ω is magnitude peak of *y*-axis gyroscope output during a gait cycle.

### 4.2. A Novel Heading Correction algorithm

**Figure 7:**
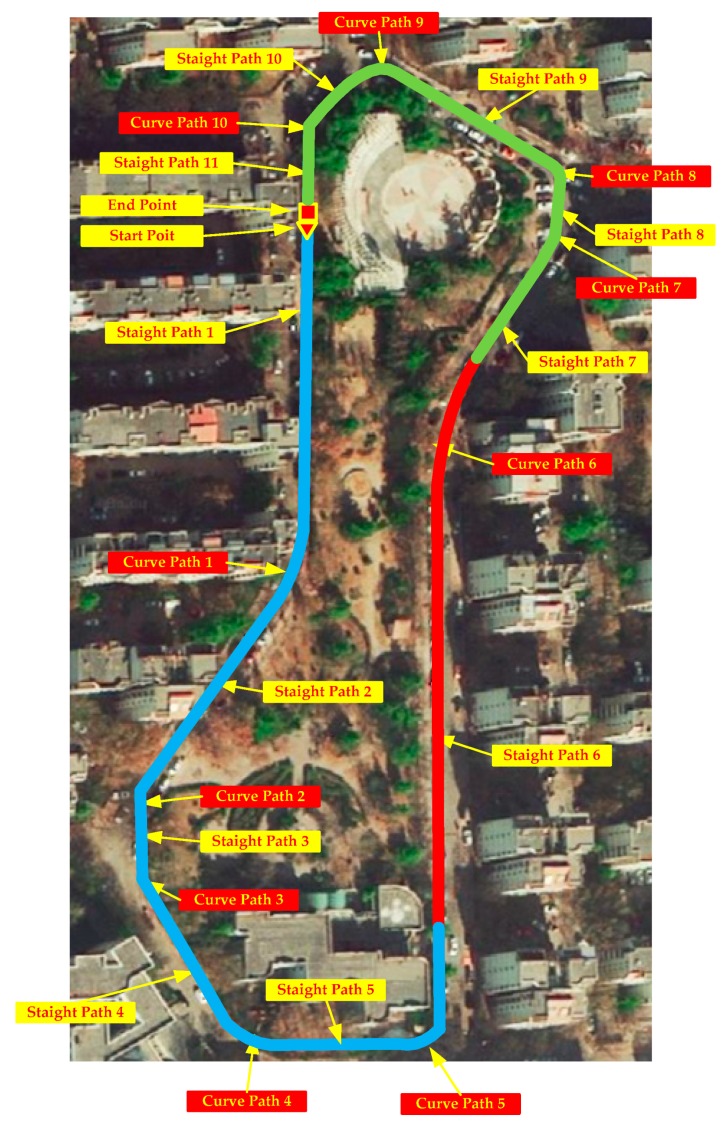
The complex irregular path is located in Shimen Community, Shijiazhuang City, Hebei Province, with 11 straight path segments and 10 curved path segments.

The original HDE and its improved algorithm iHDE can work very well in the straight path with pre-defined four or eight dominant directions. However, there are many irregular paths that are difficult for us to pre-define dominant directions for all straight paths, a typical irregular path with non-ideal oriented angle is used to describe the proposed algorithm as shown in Figure 7. To solve this problem, we propose a novel heading correction algorithm that although similar to the [27] implementation includes a more strict straight-line paths detection method and a temporary dominant direction construction method. The total dimensions of the circuit and of each segment are shown in Table 2.

A three-stride straight walking detection method was introduced to detect whether a pedestrian was walking straight or not. A pedestrian starts from the starting point of *straight path 1* in Figure 7, the average value of the initial three-stride direction was used to establish the initial dominant direction $\varphi_{s1}$ along the *straight path 1* or a total station could be used to calibrate the initial dominant direction. If the proposed algorithm detects that the pedestrian starts to walk along a curve path, such as *curve path 1*, the heading correction stops working. When the proposed algorithm detects again that the pedestrian walks along a straight path, such as the *straight path 2*, a temporary dominant direction $\varphi_{s2}$ is established. Different from the establishment method of the initial dominant direction, the subsequent dominant directions are jointly given by the dominant direction of last straight path and the direction change of the curved path as shown in Equation (37).

The detailed process of heading error calculation is as below:

(1) *Stride Direction*: The stride direction of a pedestrian is:

(32) $$\theta_{s}\left(k\right)\;=\;\arctan\left(\frac{P_{y}^{k}-P_{y}^{k-1}}{P_{x}^{k}-P_{x}^{k-1}}\right)$$

where the position $P^{k}$ is calculated using the IEZ, *k* is the index of the *k*th step.

(2) *The strict straight-line paths detection (SSLPD)*

Human walking includes straight walking and curved walking. The curved walking can be divided into fast turning and slow turning, where the fast turning refers to the motion that a person can complete through a small number of strides as shown in *curved path 2* and the difference between two consecutive strides direction is large. The slow turning is a movement that a person completes through more steps, lasting for a longer time and the difference between two consecutive strides direction is small as shown in *curved path 1*. When a person is walking along a straight path, there is a small difference between the two consecutive strides direction due to the body swing, which is similar to the phenomenon of slow turning. Therefore, it is necessary for us to develop a very strict straight-line walking detection method to distinguish between slow turning motion and straight-line swing motion.

In order to detect a trajectory as straight, we used at least three user strides. A binary parameter is computed as:

(33) $$C_{1}\left(k\right)\;=\;\{\begin{array}{cc} 1 & \max\left(\left|\theta_{s}\left(j\right)-mean\left(\theta_{s}\left(j\right)\right)\right|\right)<Th_{\theta }\\ & for\;j\;=\;k:k-2\;\\ 0 & otherwise \end{array}$$

where the $Th_{\theta }$ is an angular threshold. If $C_{1}\left(k\right)$ is large enough (above $Th_{\theta }$), it is assuming a turning motion. If not, then $C_{2}\left(k\right)$ and $C_{3}\left(k\right)$ are computed, as:

(34) $$C_{2}\left(k\right)\;=\;symbol_{back}\left(\Delta \theta_{s}\left(k-1\right)\right)\&symbol_{back}\left(\Delta \theta_{s}\left(k\right)\right)\&symbol_{back}\left(\Delta \theta_{s}\left(k+1\right)\right)$$

(35) $$C_{3}\left(k\right)\;=\;\{\begin{array}{cc} 1 & {Sum}_{\Delta \theta_{s}}\;=\;\left(\Delta \theta_{s}\left(k-1\right)+\Delta \theta_{s}\left(k\right)+\Delta \theta_{s}\left(k+1\right)\right)>Th_{\theta }^{\prime }\\ 0 & otherwise \end{array}$$

(36) $$\mathrm{SSLPD}(k)\;=\;C_{2}\left(k\right)\&C_{3}\left(k\right)$$

where $\Delta \theta_{s}\left(k\right)\;=\;\theta_{s}\left(k\right)-\theta_{s}\left(k-1\right)$. If *x* is positive, the function symbol_back(x) returns 1, or returns 0; $C_{2}\left(k\right)$ denotes the walking trend of three consecutive steps, if $C_{2}\left(k\right)$ is true, it indicates that the pedestrian is walking in the same direction. $C_{3}\left(k\right)$ denotes whether the orientation change of the three consecutive steps is large enough (above $Th_{\theta }^{\prime }$). If SSLPD(*k*) is true, it is assuming a turning motion, otherwise, it is assuming a straight walking motion. The novel heading correction method works when more than three consecutive straight walking steps are detected.

(3) *The orientation change of the curved path.* If the pedestrian is walking along a curved path detected by SSLPD method, the cumulated heading change $\Delta \theta_{curved}$ is calculated.

(4) *Establishing temporary dominant direction in real time*. If the pedestrian is walking along a straight path again, the heading of the straight path is calculated as:

(37) $$\{\begin{array}{ll} \theta_{TD}\left(n\right)\;=\;\frac{1}{3}\sum_{j\;=\;1}^{3}{\bar{\varphi }}_{n,j}, & n\;=\;1\\ \theta_{TD}\left(n\right)\;=\;\theta_{TD}\left(n-1\right)+\Delta \theta_{curve}\left(n-1\right), & n>1 \end{array}$$

where *n* denotes the *n*th detected straight-line path. $\theta_{TD}\left(n\right)$ denotes the dominant direction of the *straight path n*. ${\bar{\varphi }}_{1,j}$ is the heading average of the still-phase of the *j*-th step on the *straight path 1*. $\Delta \theta_{curve}\left(n-1\right)$ is the orientation change of the *curved path n − 1*. If n > 1, the dominant direction of *the straight path n* is equal to the sum of the dominant direction of the *straight path n − 1* and the orientation change of the *curved path n − 1*.

(5) *Is it dominant direction?* A pre-defined threshold is used to detect weather the pedestrian is walking along a dominant direction path or not. If the absolute value of the difference between the current stride direction and one of the dominant directions is small enough (below the given threshold), it is determined to be that the pedestrian is walking along the dominant direction and then the closed dominant direction is used to estimate the error in heading.

(6) The error in heading

The error in heading is calculated as:

(38) $$\delta \varphi_{m}\left(k\right)\;=\;\varphi_{m}\left(k\right)-\;\theta_{TD}\left(m\right)$$

where $\varphi_{m}\left(k\right)$ is the heading at the current sample k computed as $\varphi_{m}\left(k\right)\;=\;\arctan\left(C_{b_{k/k}}^{n}\left(2,1\right),C_{b_{k/k}}^{n}\left(1,1\right)\right)$ on the straight path m.

## 5. Experiment Validation

**Figure 8:**
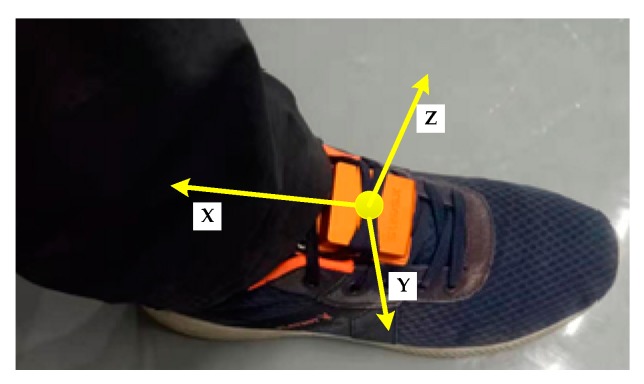
Xsens IMU mounted on the right foot.

The zero-velocity interval is very small and can be ignored when a pedestrian is running fast [11], and there is still a large positioning error in the running motion even using the adaptive still-phase detection method [15]. Therefore, this paper only addressed the still-phase detection of a pedestrian in the movement modes of walking at normal speed and running slowly. Three kind of experiments were carried out to evaluate the performance of the proposed eHDE algorithm and an IMU mounted on foot was used to collect the readings of acceleration and angular rate during experiments as shown in Figure 8. The origin is the MEMS IMU center of mass. The *x*-axis is pointing in the opposite of forward direction, the *z*-axis is pointing up vertical direction, and the *y*-axis follows the right-handed rule.

### 5.1. Performance of Adaptive Zero-Velocity Detection

**Figure 9:**
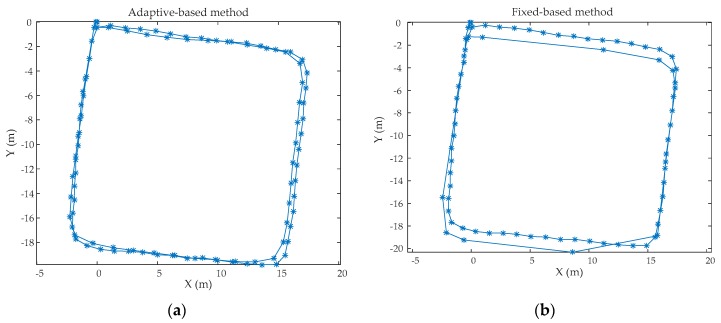
The trajectories of Person A: (**a**) the trajectories using the adaptive zero-velocity detection algorithm; (**b**) the trajectories using the fixed threshold-based algorithm.

**Figure 10:**
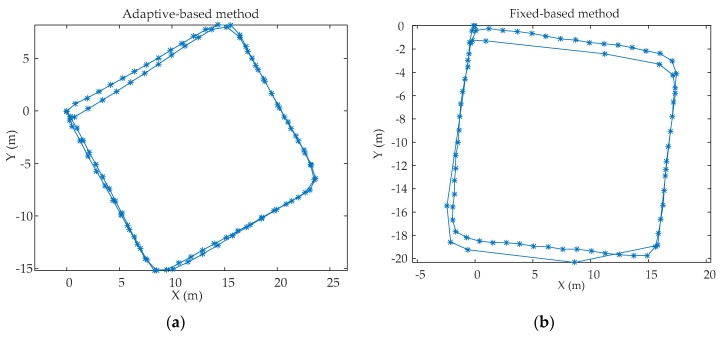
The trajectories of Person B: (**a**) the trajectories using the adaptive zero-velocity detection algorithm; (**b**) the trajectories using the fixed threshold-based algorithm.

In order to evaluate the performance of the proposed adaptive zero-velocity detection algorithm, two experiments were conducted in real environment and the size of W is 14. In the first experiment, Person A (a 32 year-old male with a height of 1.78 m and weight of 80 kg) walked along a rectangular corridor (22 m long and 22 m wide), then running slowly along the same path, each motion mode repeated for one loop. In order to verify the generality of the adaptive zero-velocity detection algorithm determined by Person A, another person, called as Person B (a 30 year old male with a height 1.80 m and a weight of 85 kg) repeated this experiment using the same adaptive zero velocity algorithm. The trajectories using the proposed adaptive zero-velocity detection method and the fixed threshold-based method were calculated. Figure 9 shows the trajectories of Person A and Figure 10 shows the trajectories of Person B. The positioning errors are shown in Table 3. It can be obviously seen that the adaptive zero-velocity detection algorithm outperforms the fixed threshold-based algorithm and the performance differs between Person A and Person B because of the uniqueness of everyone’s motion characteristics. Even so, the positioning accuracy of the adaptive zero-velocity detection method for Person B outperforms the fixed threshold-based method.

### 5.2. Performance of a Novel Heading Correction Algorithm

**Figure 11:**
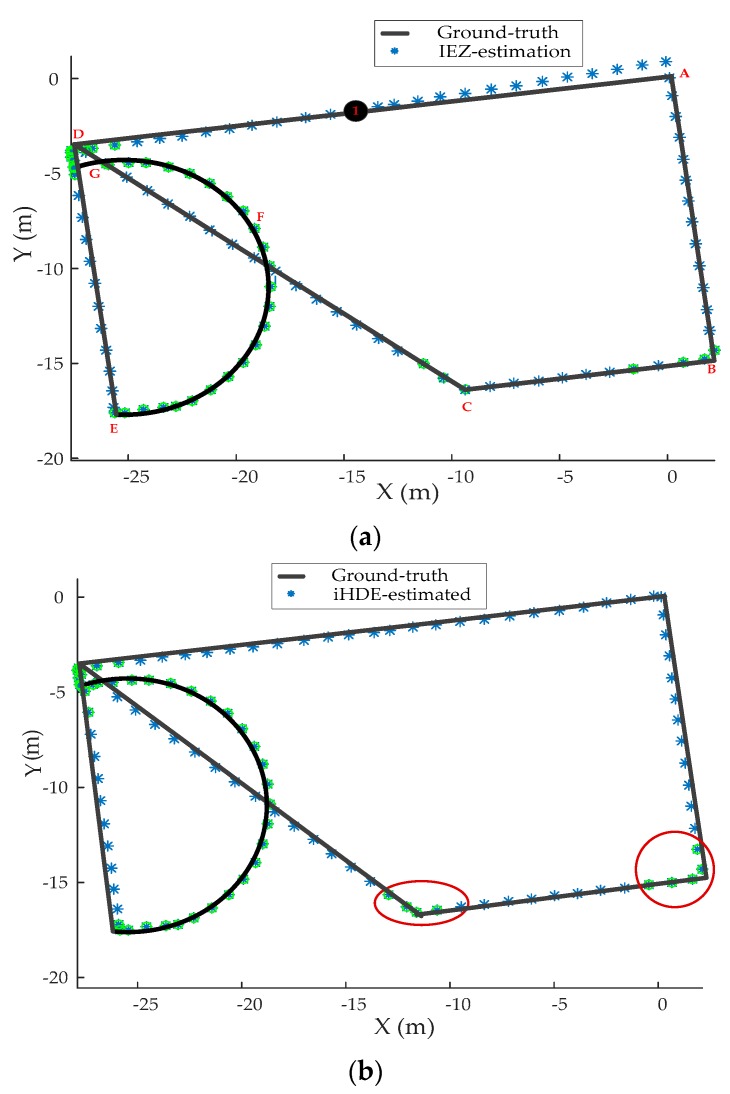

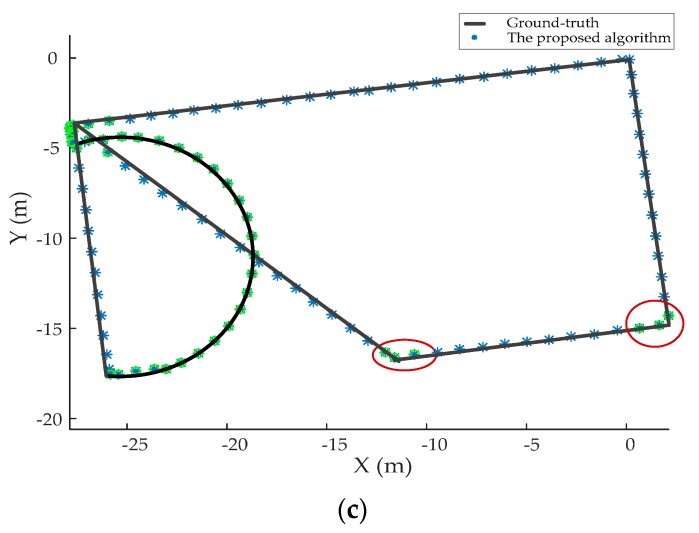
Evaluation of the algorithms. (**a**) Estimated trajectory using the IEZ algorithm. (**b**) Estimated trajectory using the iHDE algorithm. (**c**) Estimated trajectory using the proposed heading correction algorithm. The green circles represent the curved walking steps.

In order to prove that the proposed heading correction algorithm can achieve similar results to the iHDE algorithm, a trajectory with curved paths, pathways oriented at 90° and 45°, was generated as an “easy” one satisfying very well the iHDE assumptions. The pedestrian walking sequence was A-B-C-D-E-F-G-D-A, approximately 90 m. For better demonstrating the performance of the proposed algorithm in reducing the cumulated errors in the heading, we ignored the initial heading error of the IEZ algorithm. The results are shown in Figure 11. We can observe in Figure 11a that the positioning accuracy of the IEZ estimation standalone diverged from point 1. Comparing Figure 11b with Figure 11c, we can see that the proposed heading correction algorithm performed similar to iHDE, which met our expectation, because the proposed algorithm worked in a similar way as the iHDE algorithm when the path included only curved corridors and pathways oriented at 90° and 45°. This is, both of them can work in the pre-defined dominant directions and do not work in curved paths. The iHDE algorithm uses five user strides to detect whether the pedestrian is walking straight or not, while our algorithm uses three user strides which can avoid missing detection of straight strides as shown in the red oval.

**Figure 12:**
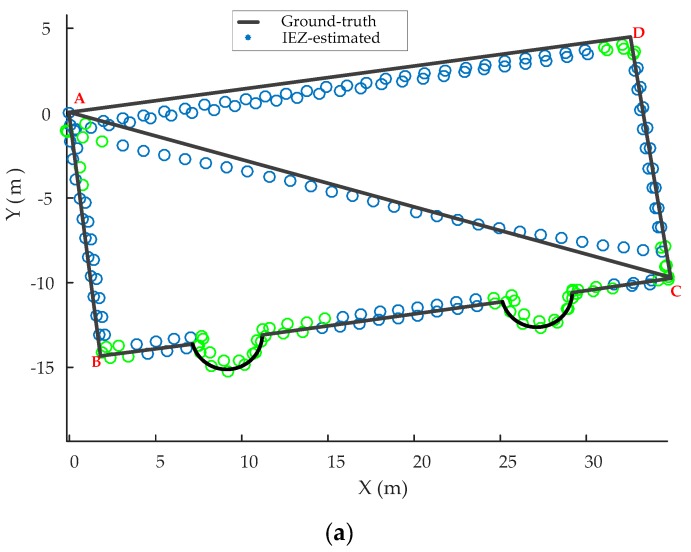

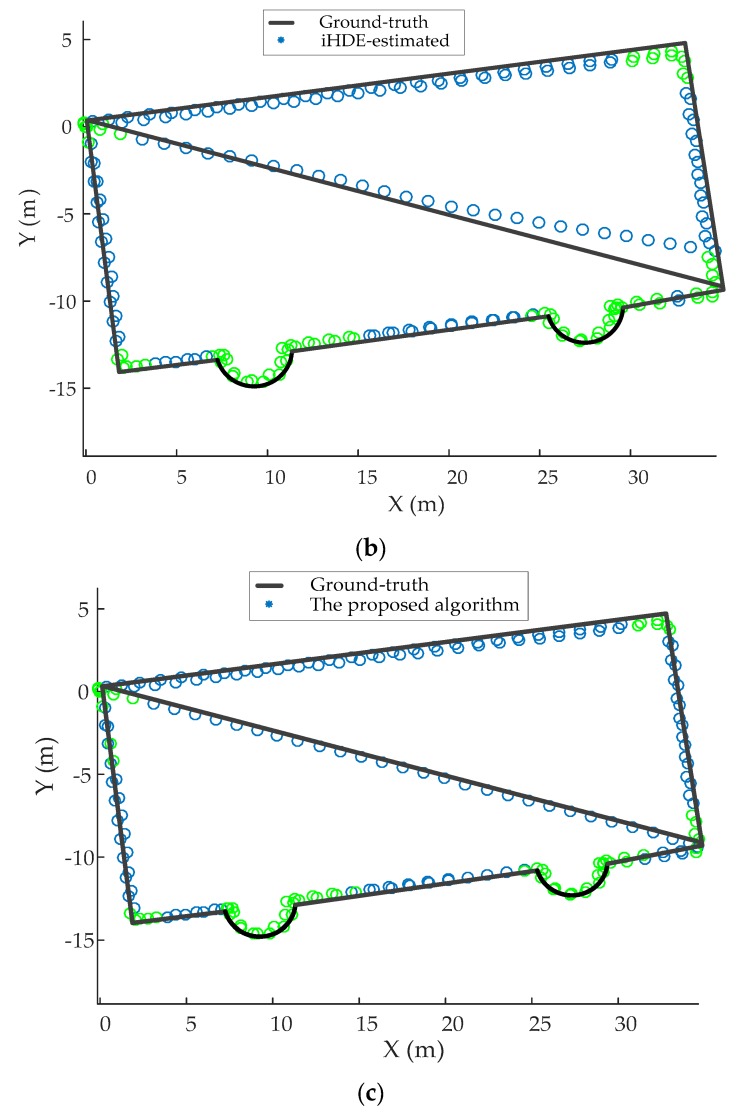
Evaluation of the algorithms. (**a**) Estimated trajectory using the IEZ method. (**b**) Estimated trajectory using the iHDE method. (**c**) Estimated trajectory using the proposed heading correction method. The green circles represent the curved walking steps.

In order to verify that the proposed heading correction algorithm outperformed iHDE, other experiments were carried out and a trajectory with a curved path, straight paths oriented at 90°, and straight paths oriented at non-ideal angles were selected as the experimental path. The walking sequence of the pedestrian was A-B-C-D-A-B-C-D-C, a total length of about 240 m. The results are shown in Figure 12. As can be seen from Figure 12a, the pedestrian trajectory gradually deviated from real trajectory using the IEZ algorithm from point D. Although the iHDE algorithm can eliminate the accumulated error in the heading in the straight paths of the A-B-C-D segments with the pre-defined dominant directions, it cannot work in the non-dominant straight path of the A-C segment as shown in Figure 12b. As can be seen from Figure 12c, the accumulated error in the heading of the straight path of A-C segment was effectively eliminated, because our proposed heading correction algorithm can establish a temporary dominant direction for the straight path of A-C segment when it is detected that the pedestrian is walking straight along the path of A-C segment. The pre-defined dominant directions are shown in Table 4. The dominant direction of the A-C straight path segment was calculated as 105.2°.

### 5.3. Performance of eHDE Algorithm

**Figure 13:**
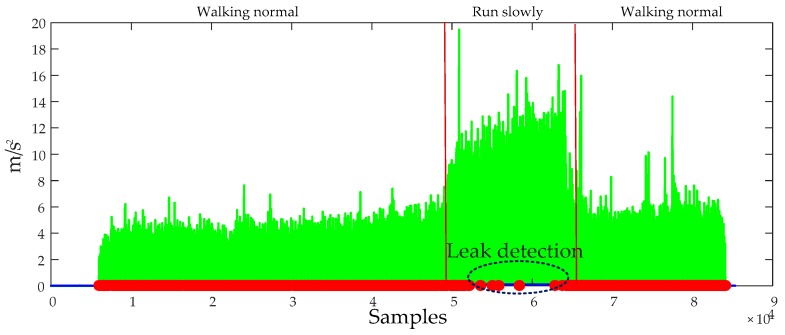
The result of the still-phase detection using $\;\delta_{\mathrm{ZUPT},1}$ with only 406 steps detected. The green lines denote the standard deviation of acceleration and the red dots denote the detected steps.

**Figure 14:**
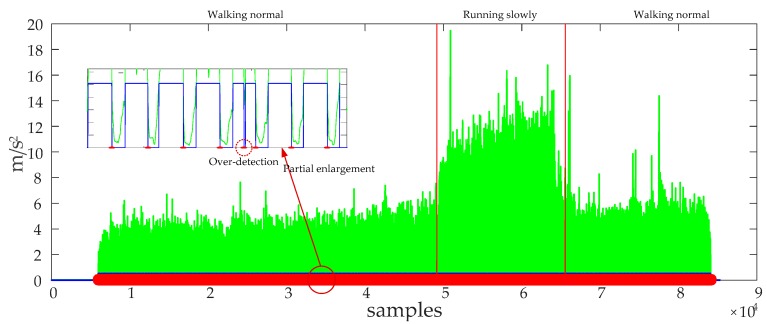
The result of the still-phase detection using $\delta_{\mathrm{ZUPT},2}$ with 598 steps detected. The green lines denote the standard deviation of acceleration and the red dots denote the detected steps.

**Figure 15:**
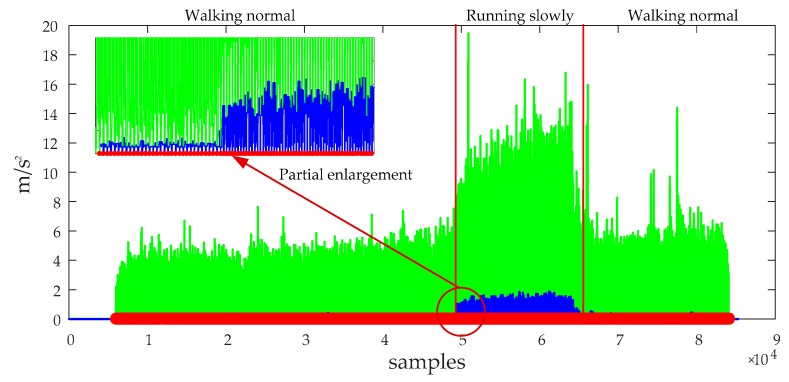
The result of the still-phase detection using adaptive still-phase detection with 517 steps detected. The green lines denote the standard deviation of acceleration and the red dots denote the detected steps.

In order to adequately demonstrate the performance of the eHDE algorithm, a complex trajectory for which it is difficult to pre-define the dominant directions was used for experiments as shown in Figure 7. The pedestrian walked a circle along the complex path in different motion modes. First, he walked at a normal speed for a distance as shown in the blue line segment in Figure 7, then ran slowly for a distance as shown in the red line segment in Figure 7, finally, he walked again at normal speed for a distance as shown in the green line segment in Figure 7 with a total 517 steps. The performance of the eHDE algorithm was evaluated in two stages: we first evaluated the performance of the adaptive ZUPT algorithm by comparing with the fixed threshold-based ZUPT algorithm and then applying the novel heading correction algorithm proposed in this paper and iHDE algorithm, respectively, to validate its effectiveness in complex paths with many straight features. Figure 13 shows that the still-phase and steps can be accurately detected using the pre-defined threshold $TH_{\mathrm{ZUPT},1}$ when the pedestrian walked at a normal speed. But, when the pedestrian ran slowly, the standard deviation of the acceleration became larger which led to the leak detection of the still-phase and steps. Figure 14 shows that although the leak detection of the steps can be avoided using a larger pre-defined threshold $TH_{\mathrm{ZUPT},2}$, it leads to the over-detection of steps. Figure 15 shows that the adaptive still-phase detection algorithm can adaptively adjust the threshold according to the change of the acceleration standard deviation.

**Figure 16:**
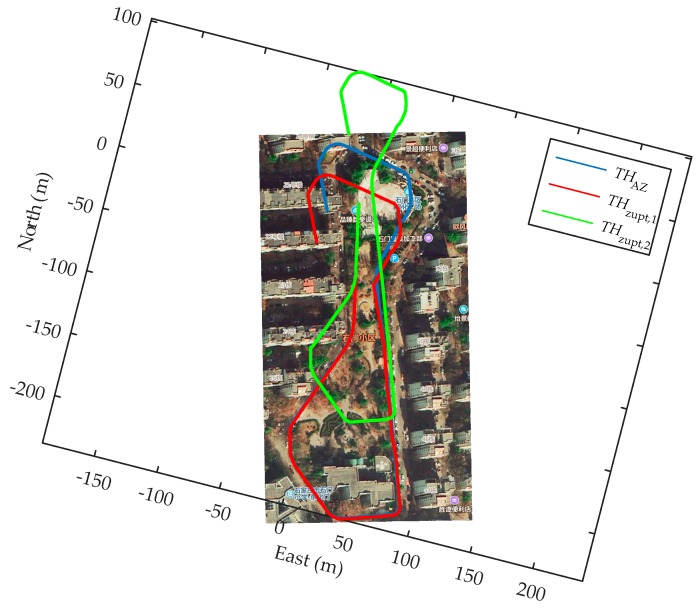
Comparison of the resulting 2D trajectories using adaptive and fixed threshold-based ZUPT methods.

The trajectories were rotated on an angle to eliminate the initial heading error inherent in the IEZ algorithm and were placed on Google Map as shown in Figure 16. As can be seen, the trajectory derived by AZUPT algorithm was closest to the true path compared with the trajectories derived by the fixed threshold-based ZUPT algorithm.

**Figure 17:**
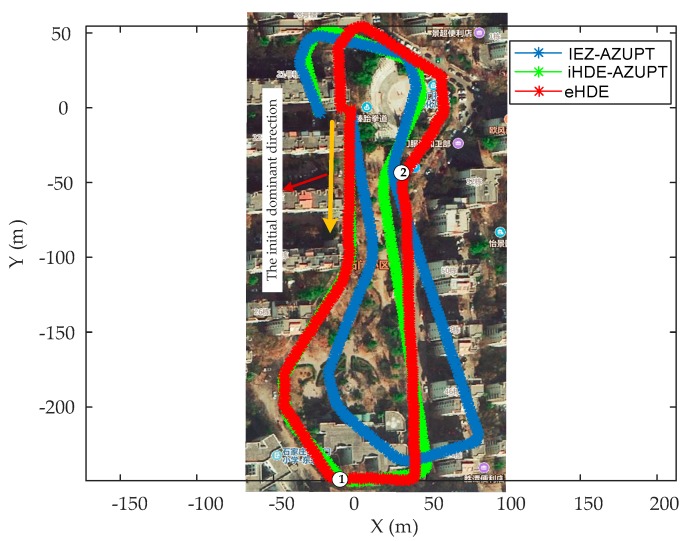
Comparison of the resulting 2D trajectories using the eHDE, iHDE–AZUPT, and IEZ–AZUPT algorithms.

In order to prove that the proposed heading correction method in eHDE was more effective than the iHDE algorithm, we combined the AZUPT with the iHDE algorithm (iHDE–AZUPT) and then the eHDE and iHDE–AZUPT algorithms were separately used to process the collection data from the IMU mounted on the foot. The iHDE algorithm requires four or eight dominant directions to be pre-defined in advance. However, it is difficult for us to determine whether the angle at which two straight paths intersect are 90°/45° or not in complex irregular paths. Therefore, we were unable to pre-define the dominant directions in advance which made the iHDE algorithm unavailable. But, if we know that the pedestrian will start walking straight along a straight-line path, the dominant direction of the straight path can be pre-defined as an initial dominant direction, such as the *straight path 1* in Figure 7, and then the iHDE–AZUPT algorithm can be used. The results of the pedestrian trajectories were placed on the Google Map as shown in Figure 17. It can be seen that the pedestrian walking trajectories generated by the eHDE and iHDE–AZUPT algorithms are almost overlapping, and the positioning accuracy is almost the same at the beginning. However, when the pedestrian passes a turn and enters *straight path 2*, the heading correction part of the iHDE algorithm fails to work and degenerates into the IEZ algorithm, the cumulated error in heading derived by iHDE algorithm gradually increases, and a significant deviation occurs from the point ①, because the dominant direction can be established in real time, eHDE algorithm can still reduce the accumulated error in heading and the deviation does not generate until the point ② which is mainly caused by turning. There is an initial heading error derived by the IEZ–AZUPT algorithm shown in the blue line in Figure 17, and it can be seen that the initial dominant direction is effective in eliminating the initial error in heading when comparing the IEZ–AZUPT algorithm with the iHDE–AZUPT algorithm or thte eHDE algorithm. The location errors are shown in Table 5. It can be obviously seen that eHDE algorithm has a higher navigation accuracy and stronger adaptability than the iHDE algorithm in irregular complex paths with many straight features and 63.75% of the location errors were reduced.

**Figure 18:**
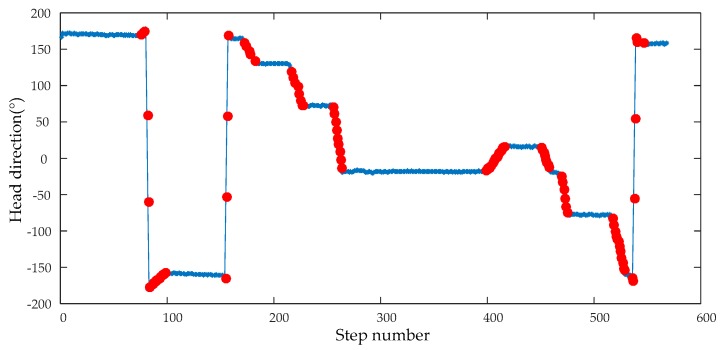
The result of the straight walking detection.

The result of the straight walking steps detection is shown in Figure 18. The blue colored star-like dots represent straight walking, and the red solid dots represent the curved walking. As can be seen, all the straight walking paths were detected.

The dominant directions of all the straight paths are shown in Table 6. The *straight path 1* and the *straight path 11* are two different straight path segments on the same straight path as shown in Figure 7 and the difference is 1.5 degree.

## 6. Conclusions

This paper presented an eHDE algorithm for pedestrian navigation with only a MEMS-IMU mounted on foot. An adaptive still-phase detection method was introduced to improve the detection accuracy of the zero-velocity interval, the relationship between the magnitude peaks of the *y*-axis gyroscope and the threshold values was established to adaptively adjust the threshold according to the motion intensity. Although the AZUPT algorithm depends on people, behavior, and many other scenarios, the performance of the AZUPT was better than the fixed threshold-based detection method. In addition, a strict straight-line path detection method was introduced and a novel heading correction method which can establish the temporary dominant direction was developed to estimate the error in heading in complex irregular paths with many straight features. The real experimental results show that the eHDE algorithm can not only improve the still-phase detection accuracy of different motion modes (walking at normal speed/running slowly) but also outperforms the iHDE algorithm in complex irregular paths with many straight features.

## Figures and Tables

**Table 1:** The optimal thresholds of different motion modes.

Level Walking/Running (rad/s)	1.96	2.82	3.68	4.50	5.0	5.5
**The threshold values**	0.13	0.20	0.45	0.6	1.0	1.4

**Table 2:** The total dimensions of the circuit and of each segment. SP is the abbreviation of straight path. CP is the abbreviation of curved path.

**Segments**	**Circuit**	**SP1**	**CP1**	**SP2**	**CP2**	**SP3**	**CP3**	**SP4**	**CP4**	**SP5**	**CP5**
Dimension (m)	689	87	27	66.5	5	16.5	7	48	15	35.23	10
**Segments**	**SP6**	**CP6**	**SP7**	**CP7**	**SP8**	**CP8**	**SP9**	**CP9**	**SP10**	**CP10**	**SP11**
Dimension (m)	170	23	43	11	13	6.5	51	15	7.5	6	26

**Table 3:** The final positioning errors.

		Positioning Error/Travelled Distance (%)	Steps
Person A	Adaptive-based method	0.67	100
	Fixed-based method	1.78	87
Person B	Adaptive-based method	1.25	92
	Fixed-based method	2.23	81

**Table 4:** The pre-defined dominant directions of the different straight path segments.

The Straight Path Segments	A-B	B-C	C-D	D-A
The pre-defined dominant directions (°)	172.5	82.5	−7.5	−97.5

**Table 5:** Location error.

Algorithms	iHDE–AZUPT	eHDE
**Location Error/Travelled Distance (%)**	2.92	1.06

**Table 6:** The dominant directions of the straight paths.

SP *n*	1	2	3	4	5	6	7	8	9	10	11
Heading (°)	−12.1	−45.7	−12.7	19.8	75.3	167.7	134.1	169.4	−134.7	−52.1	−10.6

## Abbreviations

The common abbreviations and initials in this paper.

PDR	Pedestrian Dead-Reckoning
HDE	Heuristic Drift Reduction
ZUPT	Zero Velocity Update
iHDE	Improved Heuristic Drift Elimination
eHDE	Enhanced Heuristic Drift Elimination
MEMS	Micro-Electro-Mechanical Systems
IEZ	INS-EKF-ZUPT
EKF	Extended Kalman Filter
IMU	Inertial Measurement Unit
SSLPD	Strict Straight-line Paths Detection
